# Comparative genomic analysis of primary tumors and paired brain metastases in lung cancer patients by whole exome sequencing: a pilot study

**DOI:** 10.18632/oncotarget.27837

**Published:** 2020-12-15

**Authors:** Pascale Tomasini, Fabrice Barlesi, Sophie Gilles, Isabelle Nanni-Metellus, Riccardo Soffietti, Emilie Denicolai, Eric Pellegrino, Emilie Bialecki, L’Houcine Ouafik, Philippe Metellus

**Affiliations:** ^1^Aix Marseille University, Assistance Publique Hôpitaux de Marseille, Multidisciplinary Oncology & Therapeutic Innovations Department, Marseille, France; ^2^Predictive Oncology Laboratory, Centre de Recherche en Cancérologie de Marseille, Inserm UMR1068, CNRS UMR7258, Aix-Marseille Université UM105, Marseille, France; ^3^Aix Marseille University, Assistance Publique Hôpitaux de Marseille, CHU Nord, Service de Transfert d’Oncologie Biologique, Marseille, France; ^4^Department of Neuro Oncology, University and City of Health and Science Hospital, Turin, Italy; ^5^Ramsay Santé, Hôpital Privé Clairval, Département de Neurochirurgie, Marseille, France; ^6^Aix-Marseille University, CNRS UMR 7051, Institut de Neurophysiopathologie, Marseille, France

**Keywords:** lung cancer, brain metastasis, whole exome sequencing, comparison, mutations

## Abstract

Lung cancer brain metastases (BMs) are frequent and associated with poor prognosis despite a better knowledge of lung cancer biology and the development of targeted therapies. The inconstant intracranial response to systemic treatments is partially due to tumor heterogeneity between the primary lung tumor (PLT) and BMs. There is therefore a need for a better understanding of lung cancer BMs biology to improve treatment strategies for these patients. We conducted a study of whole exome sequencing of paired BM and PLT samples. The number of somatic variants and chromosomal alterations was higher in BM samples. We identified recurrent mutations in BMs not found in PLT. Phylogenic trees and lollipop plots were designed to describe their functional impact. Among the 13 genes mutated in ≥ 1 BM, 7 were previously described to be associated with invasion process, including 3 with recurrent mutations in functional domains which may be future targets for therapy. We provide with some insights about the mechanisms leading to BMs. We found recurrent mutations in BM samples in 13 genes. Among these genes, 7 were previously described to be associated with cancer and 3 of them (*CCDC178*, *RUNX1T1*, *MUC2*) were described to be associated with the metastatic process.

## INTRODUCTION

Approximately 50% of brain metastases (BMs) are developed from lung cancers [1]. Moreover, in autopsy series, up to 50% lung cancer patients were found with BMs [2]. The occurrence of BMs during the course of lung cancer often induces poor prognosis and specific morbidity. BMs are described as the immediate cause of death in a majority of patients with BMs from solid tumors. Without any treatment, the median overall survival (OS) of patients with BMs from adenocarcinoma of the lung is short (4 to 11 weeks) [2, 3]. With treatments such as neurosurgery, stereotactic radiosurgery and whole brain radiation therapy or systemic treatments OS has improved but remains limited (6.9–13.7 months) [4]. There is thus a need for a better biological understanding of lung cancer BMs to substantially improve treatment strategies in this field.

Recently, the identification of driver oncogenes and the development of targeted therapies have improved lung cancer patients’ outcomes, especially for lung adenocarcinoma. Among the most frequent genomic alterations found in lung adenocarcinoma, *EGFR* and *BRAF* mutations, as well as *ALK* and *ROS1* rearrangements are approved biomarkers [5] as they can be targeted by approved drugs leading to an improved survival (up to 46 months) [4, 6]. However, the intracranial efficacy of targeted therapies is unpredictable. This may be explained by the presence of blood brain barrier (BBB) that limits the delivery of drugs to the brain [7], but also by tumor genomic heterogeneity [8]. In this regard there are evidences suggesting a difference in gene expression between primary lung tumors and metastases [9]. Brastianos et al. published a pioneering study comparing genomic alterations in BMs and matched primary solid tumors; and showed that genomic alterations found in primary samples of solid tumors were not representative of BMs genomic characterization [10] however, only few lung cancer samples were included. This molecular divergence may be explained by the BBB role in tumor cell migration and colonization of central nervous system [11]. This may also be explained by the hypothesis that BMs and primary lung tumors come from a common precursor but continue to evolve independently and acquire more new mutations.

Therefore, understanding lung cancer BMs biology is an actual need for better tailor treatment strategies in order to improve the outcome of patients with BMs from lung cancer.

We report here a comprehensive analysis of genomic alterations in paired primary tumors and BMs in lung cancer patients and discuss their potential implications.

## RESULTS

### Population description

A total of 9 patients (5 men and 4 women) met the eligibility criteria. The median age was 64.5 years (range 35 to 76); all but one was former or current smokers at the time of diagnosis. Finally, only 7 patients had enough quality-controlled frozen tumor tissue from both lung primary tumor and BM for whole genome sequencing. Therefore, a total of 14 samples (7 primary lung tumors and 7 paired BMs) were analyzed.

### Identified mutations

**Figure 1:**
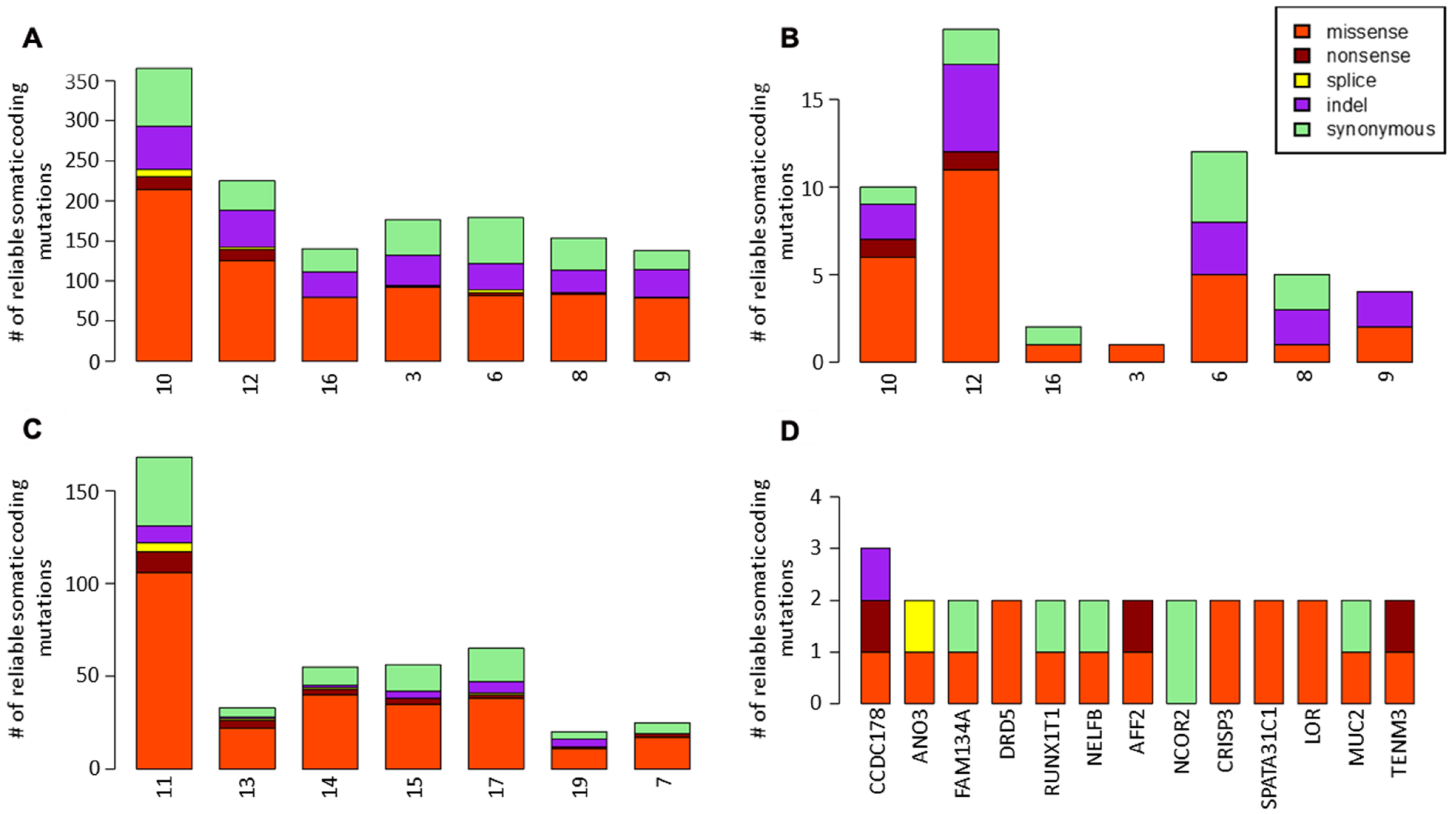
Identification of somatic variants in primary tumors and brain metastases. (**A**) single nucleotide variants (SNVs) and insertions or deletions (indels) found in primary tumors. (**B**) somatic variants specific of primary tumors. (**C**) single nucleotide variants (SNVs) and insertions or deletions (indels) found in brain metastases. (**D**) genes with recurrent mutations in brain metastases.

Identification of putatively somatic variants in primary tumors. Primary tumors had a median of 138 (range 104–311) single nucleotide variants (SNVs) and 34 (range 28–54) insertions or deletions (indels) (Figure 1A). Among these variants, 40 non-synonymous SNVs and 3 indels were found in genes that were previously described to be associated with cancers and referenced in the Cancer Gene Census of COSMIC database. A majority of putatively somatic variants found in primary tumors were also found in BM samples as 19 mutations only were found to be specific of primary tumors (Figure 1B).

### Identification of somatic variants acquired in BMs

BMs had a median of 52 (range 16–159) SNVs and 4 (range 0–9) indels acquired in comparison with primary tumor samples (Figure 1C). Among these variants, 19 non-synonymous SNVs and 1 indels were found in genes that were previously described to be associated with cancers and referenced in the Cancer Gene Census of COSMIC database. 13 genes were mutated in more than 1 BM sample (Figure 1D). The *CCDC178* gene was mutated in 3 MB samples. No driver mutation was identified among these recurrent mutations.

### Identified chromosomal alterations

**Figure 2:**
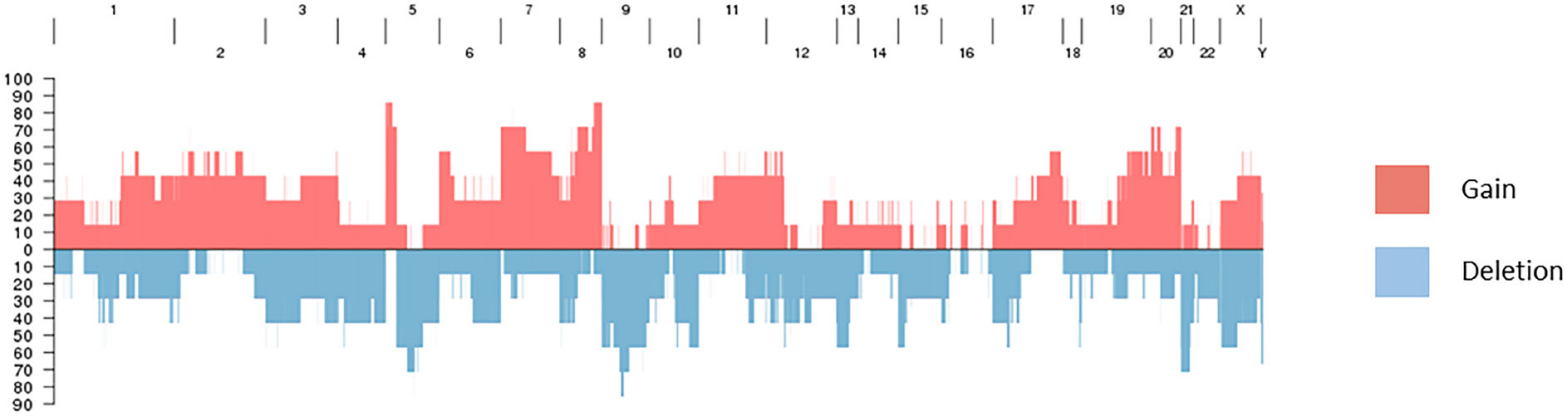
Recurrent gains and deletions in brain metastases.

Chromosomal alterations were found to be acquired by BM samples in comparison with primary tumor samples, including recurrent gains (5p, 8q) and deletions (5q, 9, 21) (Figure 2). In the 8q area is the gene *MYC*, described to be associated with cancers in the COSMIC database.

### Mutational signatures and phylogenic trees

**Figure 3:**
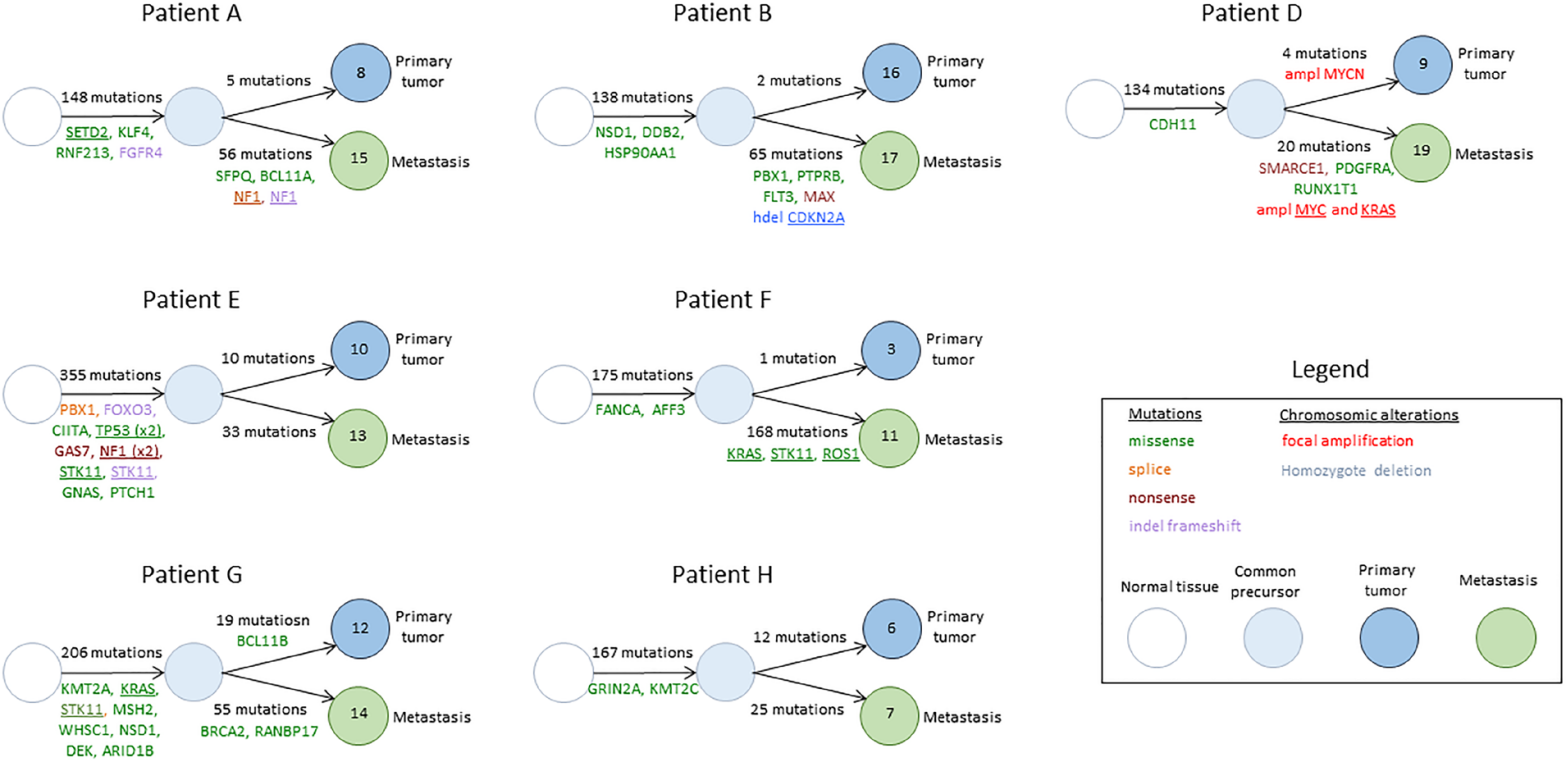
Phylogenic trees describing the common precursor between primary tumors and BMs and the number of acquired mutations in each sample. Genes mentioned were described to be associated with cancer in the COSMIC database. Underlined genes were previously described to be associated with lung cancer.

The most frequent mutations found in BMs were C>A mutations, with high transcription bias. Phylogenic trees were developed, describing the common precursor between primary tumors and BMs and the number of acquired mutations in each sample (Figure 3).

**Figure 4:**
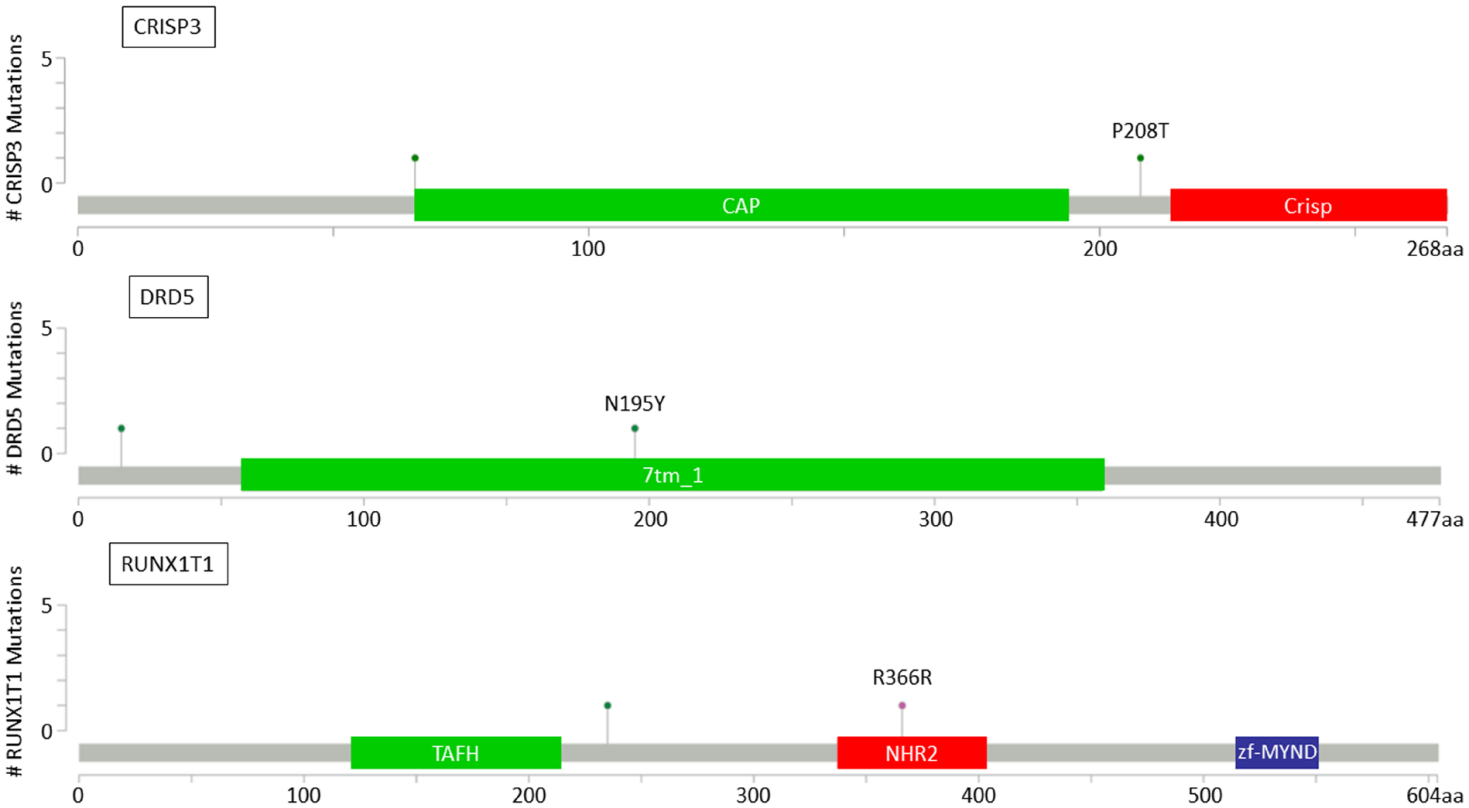
Lollipop plots for *CRISP3*, *DRD5* and *RUNX1T1* genes showing identified variants relative to a schematic representation of the gene. Colored boxes represent specific functional domains. Lollipop represents the variant identified; green lollipops stand for missense mutations and pink lollipops stand for silent mutations.

For the 13 genes identified with recurrent mutations in BM samples, lollipop plots were drawn to assess the potential impact of mutations on specific functional domains of the gene. Mutations were found in functional domains for 6 out to 13 genes (*AFF2*, *ANO3*, *CRISP3*, *DRD5*, *NELFB* and *RUNX1T1*), including 3 genes previously described to be associated with invasion and metastasis process (*CRISP3*, *DRD5*, *RUNX1T1*) (Figure 4).

## DISCUSSION

There is an urgent need for a better understanding of BMs biology in order to guide the treatment and improve the outcome of patients with BMs from solid tumors. This is especially true for lung cancer since it is the leading cause of BMs, with frequent discrepancies observed between systemic response and intracranial response to treatments. Recent data suggested that these discrepancies could be due to additional oncogenic alterations acquired in BMs in comparison with the primary tumor [10] and this hypothesis is to be further explored.

In this work, we provide with some insights about the mechanisms leading to BMs and future potential therapeutic targets. We found more mutations and chromosomal alterations in BM samples than in primary tumor samples. More than a third of these acquired mutations were found in genes previously described to be associated with cancers, including genes known to be associated with lung cancer such as *KRAS*, *ROS1* and *STK11.* Moreover, we found recurrent mutations in BM samples in 13 genes. Among these genes, 7 were previously described to be associated with cancers and 3 of them (*CCDC178*, *RUNX1T1*, *MUC2*) were described to be associated with the metastatic processes [12]. *CCDC178* was mutated in 3 samples of BMs: it was shown to be associated with the development of metastases from hepatocellular carcinoma, the regulation of the ERK pathway and anoikis resistance [12]. *RUNX1T1* mutations were shown to be predictive for the development of liver metastases from pancreatic endocrine tumors [13]. Finally, *MUC2* expression was shown to be associated with tumor differentiation and invasion in gastric cancers [14]. In addition, the dopamine receptor D5 gene (*DRD5*) was described to be involved in tumor growth inhibition via autophagic cell death [15], *NCOR2* was described to be associated with drug resistance in breast cancers [16] and *CRISP3* was associated with prostate [17] and ovarian cancers [18]. Out of these 7 genes described to be associated with invasion and metastasis, 3 were found with mutations in functional domains and have to be further explored as potential therapeutic targets.

Moreover, the frequent C>A mutations and the median 96-trinucleotide mutation profile found in BMs were consistent with previously reported signatures associated with lung cancer and tobacco smoking [19].

These results have to be validated in larger cohorts; however, these genes found to be mutated in several BM samples and associated with cancer survival or metastasis development are potential new treatment targets. For instance, *MUC2* can be targeted by a bivalent conjugate vaccine previously studied for the treatment of resistant prostate cancers [20].

The limited number of samples analyzed (14 samples from paired primary tumors and BMs of 7 patients with lung cancer) is a limitation. In addition, in these patient’s constitutional DNA was not available and the results reported here have to be compared with public databases of germline variants.

The next steps are to validate these results in larger cohorts including samples from paired primary lung tumors, BMs, extra-cranial metastases and blood in order to analyze circulating tumor DNA (ctDNA). Also, a future direction would be the comparison of ctDNA genomic alterations to those found in BMs. Liquid biopsies, including ctDNA analysis, have revolutionized the treatment of lung cancer patients since they may avoid a more invasive procedure such as tissue biopsy and some targeted drugs are approved based on the results of liquid biopsies [21].

We showed that primary lung tumor mutation profile was not representative of the BM mutation profile, with acquired mutations and chromosomic alterations in BMs. Data from literature converge to say that there are clearly some difficulties to detect ctDNA from primary brain tumors as well as from BMs in the blood [22–24]. Therefore, it will be interesting to determine whether free ctDNA analysis from cerebrospinal fluid and/or blood is more representative of BMs profile than tissue samples from the primary lung tumor.

Furthermore, functional analyses of the 13 genes mutated in several BM samples are required to validate their involvement in the process of central nervous system invasion and BM development. In addition, samples from other metastases of lung cancer should be analyzed to see whether these 13 genes are associated with the invasion and metastasis process or are specific of BMs.

In conclusion, this work on BM from lung cancer is consistent with previous data comparing genomic profile of paired BM and primary samples of solid tumors. The results reported are of interest since new potential drivers involved in the BM process could be identified. Furthermore, this study offers new hypotheses and clues for further investigation of BMs biology in order to improve treatment strategies and outcomes of patients with BMs from lung cancers.

## MATERIALS AND METHODS

### Patients’ selection

Patients were selected for whole genome sequencing of paired primary NSCLC and BM samples. The study was reviewed and approved by the human subjects Institutional Review Board of the Assistance Publique - Hôpitaux de Marseille (AP-HM).

Patients’ samples were identified from the AP-HM tumor bank (AC-2013-1786) using the electronic patient record. Selection criteria were as follows: patients above 18-year-old with histologically proven lung adenocarcinoma with BM diagnosed between 2007 and 2013, frozen samples from the primary lung tumor and brain metastasis available in the AP-HM Bio-bank. Written informed consent was obtained from all patients.

### Sample evaluation, DNA extraction and whole-exome sequencing

For all samples, pathologists reviewed HES-stained slides to estimate tumor cellularity before molecular testing. Genomic DNA (gDNA) was extracted from frozen sections using the Macherey-Nagel extraction DNA Tissue Kit (Macherey-Nagel, France) according to manufacturer’s protocol. DNA was quantified using Qubit version 2.0 (ThermoFisher Scientific, Paris, France). We performed whole-exome sequencing of extracted tissue within Integragen platform using methods as described on Illumina HiSeq [25, 26]. Samples were sequenced to median average depth of 135X.

### Identification of somatic variants in primary tumors and BMs

Raw sequence alignment and variant calling were carried out using Illumina CASAVA 1.8 software. The Ensembl Variant Effect Predictor (VEP) [27] tool was used to retrieve extensive annotations for each variant, including its presence in the 1000Genome, Exome Variant Server (EVS), ExAC or Integragen databases, its consequence on the protein sequence (synonymous, missense, nonsense, splice variant, frameshift or in-frame indels) and its functional impact.

Stringent quality controls were applied to keep only reliable variants sequenced in ≥ 10 reads, with ≥ 3 variant calls, a proportion of variant calls ≥ 15% and a QPHRED score ≥ 20 for both SNP detection and genotype calling (≥ 30 for indels). In the absence of matched non-tumor sample, we used public databases of germline variants (1000Genome, EVS, ExAC) to identify likely somatic variants. All variants referenced with a frequency ≥ 10^-5^ were excluded. Putative somatic variants in the primary lung tumor were considered to be also present in the metastasis if they were present in ≥ 2 variant calls representing ≥ 5% of the metastasis reads, and specific to the primary lung tumor otherwise. A variant was considered to be specific to the metastasis if it was seen in ≥ 3 metastatic reads (representing ≥ 15% of all reads at that position) and ≤ 1 read in the primary lung tumor (representing < 5% of all reads at that position).

### Chromosomal alterations

To identify copy-number alterations (CNAs) acquired in metastatic samples, we calculated the log ratio of the coverage in each metastasis as compared with the primary tumor of the same patient. Log-ratio profiles were then smoothed using the circular binary segmentation algorithm as implemented in the Bioconductor package *DNAcopy* [28]. The most frequent smoothed value was considered to be the zero level of each sample. Segments with a smoothed log ratio above zero + 0.15 or below zero − 0.15 were considered to have gains and deletions, respectively. Segments with a smoothed log ratio above zero + 2 or below zero − 2 were considered to have high-level amplifications and homozygous deletions, respectively.

### Mutational signature analysis and phylogenic trees

To identify mutational signatures, we classified each somatic mutation according to the 6 substitution types, taking into account the bases located directly in 5′ and 3′ of the mutated base, as previously described [19]. We used an adaptation of the R package Somatic Signatures and the Genomic Ranges package to retrieve the nucleotide context of each mutation. We performed a principal component analysis and a hierarchical clustering (cosine distance, Ward linkage) to classify tumor samples according to their 96-trinucleotide mutation spectrums. Finally, in order to assess the variant distribution per gene and the potential functional impact of mutations, lollipop plots were drawn using cBioPortal.
